# Association of urinary bisphenol A with hyperlipidemia and all-cause mortality: NHANES 2003–2016

**DOI:** 10.1371/journal.pone.0304516

**Published:** 2024-07-01

**Authors:** Lijuan Guo, Pin Zhao, Shilong Xue, Zhaowei Zhu

**Affiliations:** 1 Department of Disease Prevention and Control, The First Affiliated Hospital of Zhengzhou University, Zhengzhou, China; 2 Department of Urology, The First Affiliated Hospital of Zhengzhou University, Zhengzhou, China; Sultan Qaboos University College of Medicine and Health Science, OMAN

## Abstract

**Background:**

The connection between urinary bisphenol A (BPA) and hyperlipidemia is still unclear, and few studies have evaluated whether urinary BPA affects mortality among individuals with hyperlipidemia. Therefore, we aimed to investigate the link between urinary BPA and hyperlipidemia and assess the impact of urinary BPA on mortality risk in subjects with hyperlipidemia.

**Methods:**

We analyzed data of the National Health and Nutrition Examination Survey from 2003 to 2016. Multivariable logistic analysis was performed to examine the relationship between urinary BPA and hyperlipidemia. Cox regression analysis was carried out to investigate the relationship between urinary BPA and all-cause mortality in subjects with hyperlipidemia.

**Results:**

This study included 8,983 participants, of whom 6,317 (70.3%) were diagnosed with hyperlipidemia. The results showed that urinary BPA was higher in participants with hyperlipidemia group than those without hyperlipidemia (3.87 ± 0.32 vs. 2.98 ± 0.14, P = 0.01). Urinary BPA levels were analyzed in tertiles. Compared with tertile 1 of BPA (reference), the odds ratio (95% confidence interval) of hyperlipidemia related to tertile 3 of BPA was 1.28 (1.11–1.48). The hazard ratio for all-cause death associated with the highest versus lowest tertile of urinary BPA was 1.20 (95% confidence interval: 1.01–1.44; P = 0.04) among participants with hyperlipidemia.

**Conclusions:**

The study indicated a positive relationship between urinary BPA and the risk of hyperlipidemia. Urinary BPA was associated with a significantly higher risk of all-cause mortality in adults with hyperlipidemia.

## Introduction

Bisphenol A (BPA) is a chemical compound widely used in the production of polycarbonate plastics and epoxy resins [1]. BPA is present in various everyday products, such as toys, paper products, drinking water pipes, and building materials [2]. While BPA is known for its rapid metabolism and primary elimination through urine, its widespread incorporation into daily products suggests the potential for cumulative exposure, eliciting concerns about the long-term health implications [3]. As an extensively distributed environmental pollutant, BPA is connected to adverse effects on human health and a decrease in life expectancy consequent to chronic exposure [2].

Hyperlipidemia constitutes a cluster of disorders in lipid metabolism, commonly identified by elevated concentrations of total cholesterol (TC), triglycerides (TG), or low-density lipoprotein (LDL), and/or diminished levels of high-density lipoprotein (HDL). Almost 53% of adults in the United States have hyperlipidemia, a condition that is commonly linked to unhealthy lifestyles, including smoking, drinking, and not exercising. Hyperlipidemia stands as a firmly established risk factor for cardiovascular diseases [4] and ranks among the primary contributors to global disability-adjusted life-years, thereby substantially elevating the overall cost of healthcare [5].

A growing body of evidence from epidemiological and experimental investigations underscores the significance of BPA exposure in the development of metabolic disorders. In a preceding study, an assessment was conducted to examine the correlation between serum BPA concentration and the risk of incident dyslipidemia in middle-elderly Chinese adults, revealing a positive association between serum BPA levels and an elevated risk of hypertriglyceridemia [6]. Dunder et al. undertook a comprehensive meta-analysis study to explore the potential correlation between urinary BPA concentration and dyslipidemia across age groups, specifically in children (≤17 years old) and adults (≥18 years old). Contrary to expectations, the study results revealed a lack of statistically significant associations between urinary BPA and the various lipid variables assessed, regardless of whether the analysis focused on pediatric or adult cohorts [7].

Earlier investigations into the relationships between BPA and dyslipidemia yielded inconsistent findings [6, 8–11]. The link between urinary BPA and hyperlipidemia remains uncertain based on the existing evidence. Moreover, no prior investigation has evaluated whether urinary BPA affects the mortality of individuals with hyperlipidemia. In light of these considerations, the present study accessed data from an expansive prospective cohort representative of the national population—the 2003–2016 National Health and Nutrition Examination Survey (NHANES) database. The overarching aim was to assess the association between urinary BPA and hyperlipidemia and to delve into the potential ramifications of urinary BPA on mortality risk among subjects exhibiting hyperlipidemia.

## Methods

### Study design and participants

NHANES, a cross-sectional program meticulously crafted to assess the overall health and nutritional status of the United States population, employs a blend of interviews and physical examinations. This methodology ensures the acquisition of comprehensive data encompassing demographics, dietary habits, medical examinations, laboratory results, and questionnaire responses. Ethical approval for the NHANES protocol was obtained from the National Centre for Health Statistics ethics review board, with written informed consent procured from all participating individuals.

**Fig 1:**
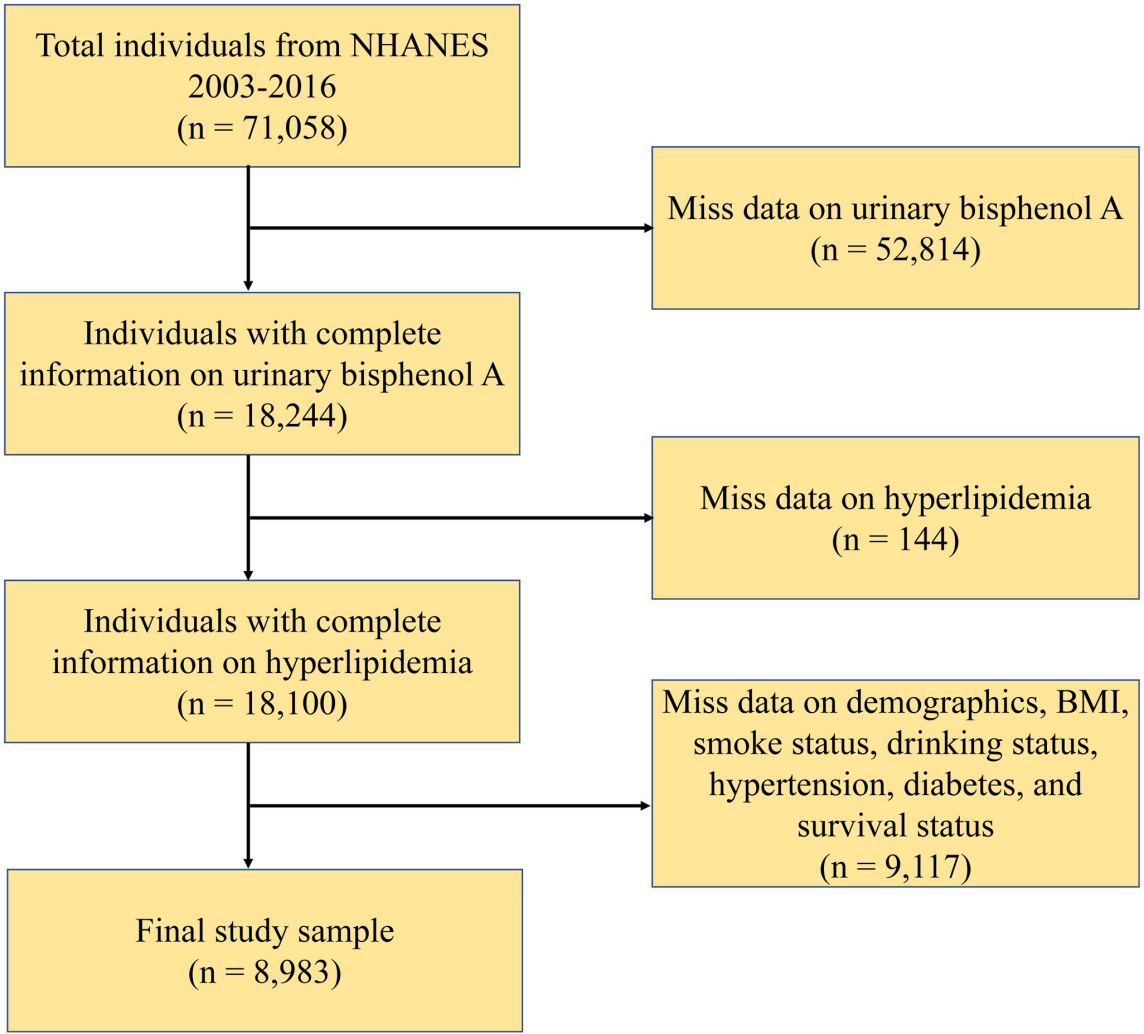
Flowchart of the study population.

In the present study, we leveraged expansive datasets encompassing seven NHANES cycles spanning from 2003–2004 to 2015–2016. The analytic population included adults who had complete information on urinary BPA, hyperlipidemia, socioeconomic and health-related characteristics. As illustrated in Fig 1, the final analytic sample comprised a total of 8,983 participants.

### Measurement of urinary BPA levels

NHANES has consistently gauged BPA concentrations in urine. In a concise depiction, the analytical procedure involved online solid-phase extraction in tandem with high-performance liquid chromatography and tandem mass spectrometry. The determination of BPA concentrations utilized spot-sample urine.

### Outcome variable

NHANES laboratory procedures and manual were utilized for the assessment of serum concentrations of TC, TG, HDL and LDL. Hyperlipidemia was characterized by meeting any of the following criteria: a documented diagnosis of elevated lipid profiles, TC ≥ 200 mg/dL, HDL < 40 mg/dL, TG ≥ 150 mg/dL, or LDL ≥ 130 mg/dL.

### Ascertainment of mortality

The NCHS database offers NHANES public-use linked mortality files up to December 31, 2019, which were cross-referenced with the National Death Index. To confirm survival status, a comprehensive set of 12 characteristics was employed to establish connections between NHANES samples and the National Death Index. For participants who survived, the survival time (in months) was calculated from the interview date to the date of death or the conclusion of the follow-up period (December 31, 2019). Our analytical approach focused on the computation of all-cause mortality.

### Covariate assessment

Control variables that may act as confounding factors in this study included socioeconomic and health-related factors. The socioeconomic parameters considered in this study included sex, age, race, education level, and marital status. Health-related features encompassed body mass index (BMI), smoking status, and alcohol consumption. Regarding smoking status, participants were divided into the following three groups: (1) never: who smoked less than 100 cigarettes in life; (2) former: who smoked more than 100 cigarettes in life and smoke not at all now; (3) now: who smoked moth than 100 cigarettes in life and smoke some days or every day. Based on alcohol consumption, participants were divided into the following three groups: (1) never: who had < 12 drinks in lifetime; (2) former: who had ≥ 12 drinks in one year and did not drink last year, or did not drink last year but drank ≥12 drinks in lifetime; (3) now: who had ≥12 drinks in one year and drink some days or every day.

Hypertension was delineated as exhibiting a mean systolic blood pressure (BP) ≥ 140 mmHg, a mean diastolic BP ≥ 90 mmHg, or being under antihypertensive medication. Diabetes was characterized by having a diagnosed condition of diabetes, current use of glucose-lowering medication, or meeting criteria such as HbA1c ≥ 6.5%, fasting serum glucose level ≥ 7.0 mmol/L, or random blood glucose ≥ 11.1 mmol/L. Impaired Fasting Glycaemia (IFG) was defined as fasting plasma glucose levels from 100 to 125 mg/dL (from 5.6 to 6.9 mmol/L) and Impaired Glucose Tolerance (IGT) as 2-hour plasma glucose levels during 75-g oral glucose tolerance test from 140 to 199 mg/dL (from 7.8 to 11.0 mmol/L) [12].

### Statistical analysis

In light of NHANES’ intricate, multistage, stratified, cluster sampling design, the data analysis process employed suitable weighting procedures to derive nationally representative estimates [13]. Baseline demographic and clinical characteristics were succinctly summarized and stratified based on hyperlipidemia status. Weights were created in NHANES to account for the complex survey design (including oversampling), survey non-response, and post-stratification adjustment to match total population counts from the Census Bureau (https://wwwn.cdc.gov/nchs/nhanes/tutorials/Weighting.aspx). Weighted means ± standard errors were used to present continuous variables, while unweighted counts with weighted percentages were employed for categorical variables. Comparative analyses between different groups involved the use of weighted t-tests for continuous variables and chi-square tests for categorical variables.

Multivariate logistic regression models were employed to determine odds ratios (ORs) and their corresponding 95% confidence intervals (CIs), assessing the link between urinary BPA and hyperlipidemia while accounting for sociodemographic covariates. Model 1 involved adjustments for sex, age, race, education level, and marital status. Model 2 expanded these adjustments to include BMI, smoking status, and drinking status. Model 3, in a more intricate analysis, further adjusted for hypertension and diabetes.

Multivariate Cox proportional hazards regression models were utilized to calculate hazard ratios (HRs) and their associated 95% CIs in order to examine the association between urinary BPA and mortality across three distinct models. Model 1 involved adjustments for sex, age, race, education level, and marital status. Model 2 extended these adjustments to include BMI, smoking status, and drinking status. Model 3, in a more comprehensive approach, further adjusted for hypertension and diabetes.

The statistical analyses were conducted using R (version 4.2.2). A two-sided P value < 0.05 was considered indicative of statistical significance.

## Results

### Population characteristics between groups

The inclusion and exclusion criteria of the current study are outlined in Fig 1. As illustrated in Fig 1, a total of 8,983 participants were enrolled in this study. Table 1 provides an overview of the characteristics of participants, categorized by hyperlipidemia status. It is noteworthy that all participants in the study were aged 20 years or older. Subjects with hyperlipidemia had a mean age of 50.62 years, which was higher than those without hyperlipidemia (40.18 years). Furthermore, individuals with hyperlipidemia demonstrated a higher BMI compared to those without hyperlipidemia (29.71 vs. 26.94, P < 0.0001). Notably, participants in the hyperlipidemia group tended to have higher urinary BPA levels (3.87 vs. 2.98, P = 0.01). The prevalence of hyperlipidemia was observed to be higher in older participants and in those with a history of smoking and drink consumption, as well as among patients with diabetes and hypertension.

**Table 1:** General characteristics of individuals included in the NHANES survey between 1999 and 2018 stratified by the presence or absence of hyperlipidemia.

Variable	Total n = 8,983^a^	Non-hyperlipidemia n = 2,666^a^	Hyperlipidemia n = 6,317^a^	*P* value
Age, mean ± SE	47.43 ± 0.28	40.18 ± 0.40	50.62 ± 0.30	< 0.0001
BMI, mean ± SE	28.86 ± 0.11	26.94 ± 0.16	29.71 ± 0.13	< 0.0001
BPA, mean ± SE	3.60 ± 0.23	2.98 ± 0.14	3.87 ± 0.32	0.01
Sex no. (Weighted%)				0.71
Female	4474 (50.46)	1274 (50.03)	3200 (50.64)	
Male	4509 (49.54)	1392 (49.97)	3117 (49.36)	
Age group no. (Weighted%)				< 0.0001
< = 49	4473 (55.99)	1828 (74.18)	2645 (47.99)	
50–65	2281 (25.42)	454 (16.01)	1827 (29.56)	
> = 65	2229 (18.59)	384 (9.81)	1845 (22.45)	
Race no. (Weighted%)				< 0.0001
Hispanic	766 (4.73)	205 (4.88)	561 (4.66)	
Non-Hispanic White	4167 (69.56)	1122 (65.33)	3045 (71.43)	
Non-Hispanic Black	1840 (11.09)	697 (14.62)	1143 (9.53)	
Mexican American	1540 (8.02)	418 (8.15)	1122 (7.97)	
Other	670 (6.59)	224 (7.02)	446 (6.40)	
Education no. (Weighted%)				< 0.0001
Less than high school	2328 (16.74)	591 (14.89)	1737 (17.55)	
High school or equivalent	2165 (23.89)	608 (21.42)	1557 (24.98)	
Some college or AA degree	2548 (31.30)	819 (32.20)	1729 (30.91)	
College graduate or above	1942 (28.07)	648 (31.48)	1294 (26.56)	
Marital status no. (Weighted%)				< 0.0001
Divorced	973 (9.96)	252 (8.16)	721 (10.75)	
Living with partner	648 (6.92)	234 (8.64)	414 (6.16)	
Married	4793 (58.28)	1234 (51.15)	3559 (61.42)	
Never married	1550 (16.78)	743 (27.19)	807 (12.19)	
Separated	295 (2.33)	90 (2.14)	205 (2.41)	
Widowed	724 (5.73)	113 (2.72)	611 (7.06)	
BMI category no. (Weighted%)				< 0.0001
<25	2587 (31.06)	1173 (46.90)	1414 (24.08)	
25–30	3005 (32.50)	756 (27.63)	2249 (34.65)	
> = 30	3391 (36.44)	737 (25.47)	2654 (41.27)	
Smoking status no. (Weighted%)				< 0.0001
Never	4787 (53.36)	1555 (59.27)	3232 (50.75)	
Former	2213 (24.66)	522 (19.16)	1691 (27.09)	
Now	1983 (21.98)	589 (21.58)	1394 (22.16)	
Drinking status no. (Weighted%				< 0.0001
Never	1256 (11.00)	350 (10.41)	906 (11.26)	
Former	1709 (15.56)	378 (10.83)	1331 (17.65)	
Now	6018 (73.43)	1938 (78.76)	4080 (71.09)	
Hypertension no. (Weighted%)				< 0.0001
No	5050 (61.08)	1907 (75.76)	3143 (54.61)	
Yes	3933 (38.92)	759 (24.24)	3174 (45.39)	
Diabetes no. (Weighted%)				< 0.0001
No	6596 (78.51)	2296 (89.81)	4300 (73.53)	
IGT	358 (3.48)	72 (2.17)	286 (4.06)	
IFG	392 (4.26)	64 (1.78)	328 (5.35)	
DM	1637 (13.75)	234 (6.24)	1403 (17.06)	

BMI, body mass index; BPA, bisphenol A; DM, diabetes mellitus; IFG, impaired fasting glycaemia; IGT, impaired glucose tolerance; NHANES, the National Health and Nutrition Examination Survey; SE, standard error. Note: Mean ± SE for continuous variables, the P value was calculated by weighted t test; Number (Weighted%) for categorical variables, the P value was calculated by weighted chi-square test. ^a^Unweighted number of observations in dataset.

### Relationship between urinary BPA and hyperlipidemia

The overall urinary BPA concentrations ranged from 0.14 to 965. To ensure the validity of our findings, we categorized urinary BPA into tertiles according to their levels: the first tertile group (Q1, n = 3,246, BPA = 0.14 to 1.1), the second tertile group (Q2, n = 2,833, BPA = 1.1 to 2.7), and the third tertile group (Q3, n = 2,904, BPA = 2.7 to 965). Each of the three models illustrated a positive association between urinary BPA levels and the prevalence of hyperlipidemia based on the BPA tertiles (Table 2). The logistic regression analyses showed that higher tertiles of BPA were related to a higher risk of hyperlipidemia. In model 1 (OR = 1.35; 95% CI: 1.17–1.56), model 2 (OR = 1.26; 95% CI: 1.09–1.46), and model 3 (OR = 1.28; 95% CI: 1.11–1.48), the incidence of hyperlipidemia exhibited an elevated risk in the highest tertile compared to the lowest tertile. A multivariate regression analysis also revealed that age, race, marital status and BMI were significantly related to hyperlipidemia. Currently and former smokers have a higher incidence of hyperlipidemia. The presence of hypertension (OR = 1.44; 95% CI: 1.23–1.67) and diabetes (OR = 1.67; 95% CI: 1.34–2.09) were significantly linked to an elevated risk of hyperlipidemia.

**Table 2:** Relationship between urinary bisphenol A and hyperlipidemia among adults aged 20 years or older.

Variable	Model 1		Model 2		Model 3	
	OR (95% CI)	*P* value	OR (95% CI)	*P* value	OR (95% CI)	*P* value
BPA category						
Q1	ref	ref	ref	ref	ref	ref
Q2	1.21 (1.04, 1.42)	0.01	1.12 (0.96, 1.32)	0.15	1.13 (0.96, 1.33)	0.13
Q3	1.35 (1.17, 1.56)	<0.001	1.26 (1.09, 1.46)	0.002	1.28 (1.11, 1.48)	<0.001
Sex						
Female	ref	ref	ref	ref	ref	ref
Male	1.01 (0.88, 1.16)	0.84	0.91 (0.80, 1.05)	0.19	0.88 (0.76, 1.01)	0.06
Age group						
≤49	ref	ref	ref	ref	ref	ref
50–65	2.52 (2.13, 2.99)	<0.0001	2.31 (1.93, 2.76)	<0.0001	1.96 (1.65, 2.34)	<0.0001
≥65	2.81 (2.37, 3.33)	<0.0001	2.63 (2.20, 3.15)	<0.0001	1.97 (1.63, 2.38)	<0.0001
Race						
Hispanic	ref	ref	ref	ref	ref	ref
Non-Hispanic White	0.95 (0.75, 1.20)	0.67	0.98 (0.77, 1.25)	0.87	0.99 (0.78, 1.27)	0.95
Non-Hispanic Black	0.65 (0.51, 0.82)	<0.001	0.58 (0.46, 0.74)	<0.0001	0.56 (0.44, 0.71)	<0.0001
Mexican American	0.98 (0.76, 1.28)	0.9	0.95 (0.73, 1.23)	0.68	0.96 (0.73, 1.25)	0.74
Other	0.99 (0.76, 1.30)	0.96	1.16 (0.87, 1.56)	0.31	1.13 (0.84, 1.53)	0.41
Education						
Less than high school	ref	ref	ref	ref	ref	ref
High school or equivalent	1.03 (0.86, 1.22)	0.77	1.03 (0.86, 1.22)	0.77	1.05 (0.88, 1.26)	0.56
Some college or AA degree	0.88 (0.75, 1.04)	0.12	0.91 (0.77, 1.08)	0.27	0.94 (0.79, 1.11)	0.45
College graduate or above	0.72 (0.60, 0.87)	0.001	0.89 (0.72, 1.10)	0.26	0.93 (0.75, 1.15)	0.49
Marital status						
Divorced	ref	ref	ref	ref	ref	ref
Living with partner	0.73 (0.58, 0.93)	0.01	0.77 (0.60, 0.99)	0.04	0.79 (0.61, 1.02)	0.07
Married	1.00 (0.82, 1.22)	1	1.04 (0.84, 1.29)	0.71	1.05 (0.85, 1.31)	0.63
Never married	0.50 (0.40, 0.64)	<0.0001	0.57 (0.45, 0.73)	<0.0001	0.59 (0.46, 0.76)	<0.0001
Separated	1.02 (0.71, 1.48)	0.9	1.02 (0.69, 1.52)	0.91	1.03 (0.69, 1.53)	0.89
Widowed	1.26 (0.90, 1.76)	0.18	1.34 (0.95, 1.89)	0.09	1.35 (0.95, 1.91)	0.1
BMI category						
<25			ref	ref	ref	ref
25–30			2.31 (1.97, 2.71)	<0.0001	2.20 (1.87, 2.59)	<0.0001
≥30			3.04 (2.57, 3.60)	<0.0001	2.58 (2.17, 3.06)	<0.0001
Smoking status						
Never			ref	ref	ref	ref
Former			1.21 (1.00, 1.46)	0.05	1.17 (0.97, 1.41)	0.09
Now			1.47 (1.23, 1.75)	<0.0001	1.44 (1.20, 1.72)	<0.001
Drinking status						
Never			ref	ref	ref	ref
Former			1.09 (0.87, 1.36)	0.47	1.07 (0.85, 1.35)	0.57
Now			0.88 (0.71, 1.09)	0.24	0.90 (0.72, 1.12)	0.35
Hypertension						
No					ref	ref
Yes					1.44 (1.23, 1.67)	<0.0001
Diabetes						
No					ref	ref
IGT					1.64 (1.11, 2.41)	0.01
IFG					2.47 (1.61, 3.79)	<0.0001
DM					1.67 (1.34, 2.09)	<0.0001

BMI, body mass index; BPA, bisphenol A; DM, diabetes mellitus; IFG, impaired fasting glycaemia; IGT, impaired glucose tolerance; NHANES, the National Health and Nutrition Examination Survey; SE, standard error.

Model 1, adjusted for demographic characteristics (sex, age group, race, education, marital status)

Model 2, adjusted for demographic characteristics (sex, age group, race, education, marital status); BMI category, smoking status, and drinking status

Model 3, adjusted for demographic characteristics (sex, age group, race, education, marital status); BMI category, smoking status, and drinking status; hypertension, and diabetes.

**Fig 2:**
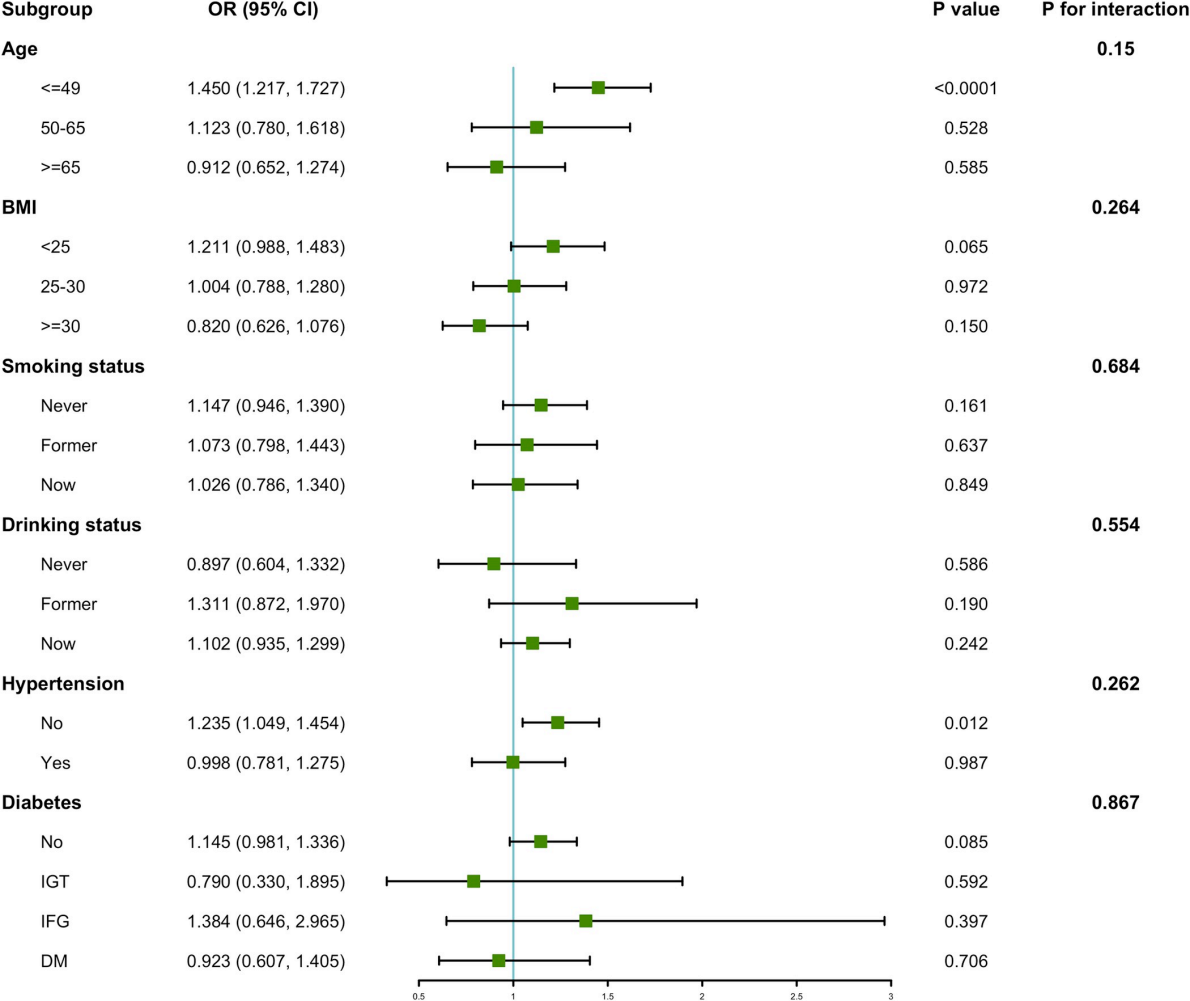
Forest plot for subgroup analysis between urinary bisphenol A and hyperlipidemia.

There was a difference in general characteristics (including age and BMI) between the hyperlipidemia group and the non-hyperlipidemia group. Thus, subgroup analyses were performed and study participants were systematically categorized based on age, BMI, smoking behaviors, alcohol usage, hypertension, and diabetes (Fig 2). Results from subgroup analyses revealed affirmative correlation between urinary bisphenol A levels and hyperlipidemia among participants aged ≤ 49 years. Similarly, a positive association was identified in those without hypertension.

### Relationship between urinary BPA and all-cause mortality in subjects with hyperlipidemia

Within the cohort, 6317 participants were diagnosed with hyperlipidemia, and a total of 990 all-cause deaths were recorded over a median follow-up period of 110 months (S1 Table). The investigation aimed to evaluate the influence of urinary BPA on the risk of all-cause mortality in individuals with hyperlipidemia. As delineated in Table 3, heightened urinary BPA levels were correlated with an increased risk of all-cause mortality in participants with hyperlipidemia, as indicated by Model 1 (HR: 1.18, 95% CI: 0.97–1.44). Nevertheless, this observed difference did not attain statistical significance (P = 0.09). Upon adjustment for sex, age, race, education level, marital status, smoking status, and drinking status in Model 2, urinary BPA levels were linked to an elevated risk of all-cause mortality in participants diagnosed with hyperlipidemia (HR: 1.23, 95% CI: 1.02–1.48, P = 0.03). This association persisted in Model 3, where additional adjustments were made for hypertension and diabetes. In this comprehensive model, the multivariable-adjusted HR for all-cause mortality associated with the highest versus lowest tertile of urinary BPA was 1.20 (95% CI: 1.01–1.44, P = 0.04) among individuals with hyperlipidemia.

**Table 3:** Association of urinary bisphenol A with all-cause mortality in individuals with hyperlipidemia among adults aged 20 years or older.

Variable	Model 1		Model 2		Model 3	
	HR (95% CI)	*P* value	HR (95% CI)	*P* value	HR (95% CI)	*P* value
BPA category						
Q1	ref	ref	ref	ref	ref	ref
Q2	1.17 (0.96, 1.41)	0.11	1.21 (1.00, 1.46)	0.05	1.21 (1.01, 1.45)	0.03
Q3	1.18 (0.97, 1.44)	0.09	1.23 (1.02, 1.48)	0.03	1.20 (1.01, 1.44)	0.04
Sex						
Female	ref	ref	ref	ref	ref	ref
Male	1.43 (1.19, 1.73)	<0.001	1.39 (1.15, 1.66)	<0.001	1.34 (1.12, 1.60)	0.001
Age group						
≤49	ref	ref	ref	ref	ref	ref
50–65	3.80 (2.65, 5.43)	<0.0001	4.02 (2.82, 5.73)	<0.0001	3.13 (2.17, 4.53)	<0.0001
≥65	16.91 (12.34,23.17)	<0.0001	18.57 (13.68,25.22)	<0.0001	12.49 (8.92,17.49)	<0.0001
Race						
Hispanic	ref	ref	ref	ref	ref	ref
Non-Hispanic White	1.53 (1.00, 2.35)	0.05	1.48 (0.97, 2.25)	0.07	1.51 (1.02, 2.23)	0.04
Non-Hispanic Black	1.33 (0.86, 2.05)	0.2	1.24 (0.80, 1.92)	0.33	1.12 (0.74, 1.71)	0.58
Mexican American	1.00 (0.68, 1.49)	0.98	1.06 (0.69, 1.62)	0.79	1.04 (0.70, 1.53)	0.85
Other	1.32 (0.70, 2.48)	0.4	1.18 (0.66, 2.13)	0.58	1.23 (0.67, 2.28)	0.51
Education						
Less than high school	ref	ref	ref	ref	ref	ref
High school or equivalent	0.75 (0.58, 0.98)	0.03	0.80 (0.61, 1.04)	0.1	0.80 (0.63, 1.04)	0.09
Some college or AA degree	0.66 (0.50, 0.87)	0.003	0.75 (0.56, 1.00)	0.05	0.75 (0.57, 0.99)	0.04
College graduate or above	0.49 (0.36, 0.66)	<0.0001	0.60 (0.44, 0.83)	0.002	0.65 (0.49, 0.88)	0.005
Marital status						
Divorced	ref	ref	ref	ref	ref	ref
Living with partner	0.74 (0.44, 1.25)	0.26	0.77 (0.47, 1.27)	0.3	0.76 (0.45, 1.26)	0.28
Married	0.63 (0.49, 0.81)	<0.001	0.72 (0.56, 0.94)	0.02	0.70 (0.54, 0.90)	0.01
Never married	0.76 (0.50, 1.14)	0.18	0.84 (0.57, 1.24)	0.38	0.81 (0.54, 1.21)	0.3
Separated	1.06 (0.57, 1.97)	0.86	1.03 (0.58, 1.86)	0.91	1.13 (0.62, 2.05)	0.69
Widowed	1.11 (0.83, 1.49)	0.49	1.26 (0.94, 1.69)	0.12	1.27 (0.95, 1.70)	0.1
BMI category						
<25			ref	ref	ref	ref
25–30			0.72 (0.57, 0.90)	0.004	0.69 (0.55, 0.86)	0.001
≥30			0.85 (0.67, 1.07)	0.15	0.66 (0.51, 0.84)	<0.001
Smoking status						
Never			ref	ref	ref	ref
Former			1.28 (1.02, 1.59)	0.03	1.21 (0.98, 1.50)	0.08
Now			2.26 (1.73, 2.94)	<0.0001	2.22 (1.74, 2.85)	<0.0001
Drinking status						
Never			ref	ref	ref	ref
Former			1.15 (0.89, 1.49)	0.28	1.19 (0.91, 1.55)	0.2
Now			0.74 (0.57, 0.96)	0.02	0.81 (0.61, 1.07)	0.14
Hypertension						
No					ref	ref
Yes					1.80 (1.45, 2.23)	<0.0001
Diabetes						
No					ref	ref
IGT					0.98 (0.70, 1.37)	0.89
IFG					1.13 (0.79, 1.61)	0.5
DM					1.83 (1.51, 2.21)	<0.0001

BMI, body mass index; BPA, bisphenol A; DM, diabetes mellitus; IFG, impaired fasting glycaemia; IGT, impaired glucose tolerance; NHANES, the National Health and Nutrition Examination Survey; SE, standard error.

Model 1, adjusted for demographic characteristics (sex, age group, race, education, marital status)

Model 2, adjusted for demographic characteristics (sex, age group, race, education, marital status); BMI category, smoking status, and drinking status

Model 3, adjusted for demographic characteristics (sex, age group, race, education, marital status); BMI category, smoking status, and drinking status; hypertension, and diabetes.

## Discussion

This study stands out as one of the largest endeavors to explore the potential relationship between urinary BPA and prevalent hyperlipidemia in adult participants, utilizing NHANES data from 2003 to 2016. Our comprehensive analysis revealed a significant association between higher urinary BPA levels and an increased risk of hyperlipidemia. Moreover, survival analysis demonstrated a substantial connection between elevated urinary BPA levels and a higher risk of all-cause mortality in adults with hyperlipidemia. These findings underscore the need for further in-depth exploration through both basic and clinical research.

Hyperlipidemia is a metabolic disorder that is prevalent in modern society and is often associated with unhealthy diets and sedentary lifestyles. It is acknowledged as one of the foremost risk factors contributing significantly to the onset of various diseases [14]. The diagnosis of hyperlipidemia is generally based on the abnormal deviation of one or more plasma lipids, including elevated levels of TG, TC, or LDL and decreased levels of HDL. The etiology of hyperlipidemia is determined by combining genetic and environmental factors, including diet, lifestyle, and even geographic location. Nonetheless, the fundamental mechanism contributing to this condition remains enigmatic. Monitoring trends in the prevalence of hyperlipidemia is essential for informing health care policy and planning. Timely prevention and management of hyperlipidemia may effectively prevent the occurrence of related diseases [15–17].

BPA is a presumed environmental endocrine disruptor widely distributed in our surroundings, primarily due to its extensive application as a monomer in the production of polycarbonate plastics and epoxy resins. Elevated levels of exposure to BPA have been associated with the potential to induce various metabolic and cardiovascular disorders. Numerous animal studies have been conducted to explore the correlation between BPA exposure and the development of metabolic disorders, particularly hyperlipidemia. Miyawaki et al. documented that prolonged exposure of mice to BPA during both perinatal and postnatal periods resulted in the manifestation of obesity and hyperlipidemia [8]. In a study by Marmugi et al., the influence of protracted BPA exposure spanning several months on hepatic and plasma metabolic markers in adult mice was examined. The investigation highlighted that sustained exposure to BPA during adulthood was associated with the development of hyperglycemia and hypercholesterolemia in mice [9]. Nevertheless, Silva and collaborators observed that maternal exposure to BPA during gestation and lactation, even at low doses, resulted in enduring alterations in the regulation of metabolic homeostasis in the offspring. This exposure influenced sex steroids and thyroid hormone levels, compromised behavior, yet did not culminate in obesity or dyslipidemia [18]. Previous studies have delved into the potential mechanisms involved in the BPA exposure induced hyperlipidemia. Fang C and colleagues observed that BPA exposure led to increased adipose accumulation and hepatic and myocardial injuries accompanied by up-regulation of endoplasmic reticulum stress and inflammatory and lipid metabolism markers in livers [19]. Another study provided the first evidence that BPA promoted cholesterol absorption in the intestinal cells and the stimulatory effect of BPA was mediated, at least in part, by sterol regulatory element binding protein-2-Niemann-Pick C1-like 1 signaling pathway [20].

In addition to the aforementioned animal studies, the findings from epidemiological investigations on the correlation between BPA exposure and hyperlipidemia exhibit inconsistencies. Notably, a pilot study reported no significant association between BPA levels and diabetes, hypertension, dyslipidemia, age, or BMI [21]. A meta-analysis covering six cycles (2003–2014) of NHANES data found no discernible associations between urinary BPA and five distinct lipid variables, across both children and adults [7]. Conversely, Li et al. explored the relationship between serum BPA concentration and the occurrence of dyslipidemia, revealing that heightened BPA exposure was associated with a higher prevalence of low-HDL cholesterolemia [22]. A recent study highlighted an association between serum BPA concentration and modifications in blood lipid levels, as well as an elevated risk of developing dyslipidemia among middle-elderly Chinese adults [6]. Thus, the regulatory role of BPA in host lipid metabolism and hyperlipidemia remains largely unquantified. Our findings indicated that urinary BPA was significantly related to higher rates of prevalent hyperlipidemia, even after adjusting for various confounding factors. These results suggest that active measures aimed at reducing urinary BPA have the potential to prevent hyperlipidemia.

The prevalence and fatality rates of hyperlipidemia and its complications are increasing rapidly, contributing to almost 50% of total deaths worldwide. The results from the study conducted by Wei X et al. imply that hyperlipidemia might exert a detrimental impact on the prolonged survival of diabetic individuals. This is supported by their observation that among peritoneal dialysis patients with diabetes, the coexistence of hyperlipidemia was associated with the highest risk of all-cause mortality [23]. Considering the high-volume industrial production of BPA, prospective investigations were conducted to scrutinize the correlation between BPA exposure and prolonged health outcomes. Notably, elevated BPA exposure demonstrated a significant association with an augmented risk of both all-cause mortality and cardiovascular mortality within a nationally representative cohort of adults in the United States [24, 25]. In the current study, we noted a significant correlation between elevated urinary BPA levels and an increased risk of all-cause mortality in individuals diagnosed with hyperlipidemia. Additionally, enhancing dietary quality emerged as a mitigating factor, demonstrating the potential to reduce the risk of both all-cause and cardiovascular mortality, particularly at low BPA exposure levels [2]. Nevertheless, given the constrained protective efficacy of dietary quality in mitigating the impact of BPA exposure, the imperative goal remains the minimization of BPA exposure [2].

As one of the most extensive investigations to date exploring the correlation between urinary BPA and hyperlipidemia, the study has several notable strengths. Firstly, our analysis utilized nationally representative survey data spanning a period of 14 years from the NHANES. Furthermore, we applied appropriate statistical procedures and sample weights to obtain prevalence estimates that accurately mirror the characteristics of the broader population. The robust sample size within the NHANES dataset enabled us to account for potential confounding variables. Secondly, the participants in the present study underwent random sampling from the general population, ensuring the representativeness and generalizability of our findings. Thus, our results can be extrapolated to the broader US population with confidence. Thirdly, the extensive sample size, high event rates, and prolonged follow-up duration of our investigation enabled a thorough examination of the correlation between urinary BPA and mortality among individuals with hyperlipidemia.

It is essential to interpret our findings in consideration of specific constraints. Firstly, the cross-sectional design inherent in the NHANES data prevents the establishment of causality between urinary BPA levels and hyperlipidemia. Secondly, it is recognized that a single urinary BPA value may not fully characterize an individual’s long-term urinary BPA levels. Thirdly, despite our efforts to adjust for relevant confounding variables, there may still be residual confounding from unmeasured or unknown factors. However, we accounted for potential survival predictors to mitigate the influence of confounding variables. Despite these constraints, our investigation unveiled a positive association between urinary BPA, hyperlipidemia and mortality. Future research with larger sample sizes and more precise measurement methods is crucial to establishing a more definitive causal connection between urinary BPA and hyperlipidemia.

## Conclusions

The investigation identified a noteworthy positive correlation between urinary BPA levels and the likelihood of hyperlipidemia. Elevated urinary BPA levels were linked to a significantly increased risk of all-cause mortality in adults diagnosed with hyperlipidemia. Minimizing BPA exposure may represent a viable strategy for preventing hyperlipidemia and lowering mortality risk, bearing potential implications for both clinical practice and public health.

## Supporting information

S1 Table Causes of death in participants with or without hyperlipidemia. (DOCX)

S1 Data Data of the manuscript. (XLSX)
