# Supplementary material for: Association of urinary bisphenol A with hyperlipidemia and all-cause mortality: NHANES 2003–2016
Source: PLoS One. 2024 Jul 1;19(7):e0304516. doi: 10.1371/journal.pone.0304516 (PMC11216755; doi:10.1371/journal.pone.0304516)
Supplement: S1 Table — (DOCX) [file pone.0304516.s001.docx]

| **Causes** | **Hyperlipidemia** | |
| --- | --- | --- |
|  | No | Yes |
| **Accidents** | 7 | 29 |
| **Ad** | 7 | 37 |
| **Brain** | 10 | 51 |
| **Cancer** | 79 | 219 |
| **Dm** | 5 | 39 |
| **Heart** | 60 | 275 |
| **Ip** | 7 | 14 |
| **Kidney** | 6 | 19 |
| **Lung** | 14 | 58 |
| **Other** | 78 | 249 |

S1 Table Causes of death in participants with or without hyperlipidemia
